# Axial length and its associations in a Russian population: The Ural Eye and Medical Study

**DOI:** 10.1371/journal.pone.0211186

**Published:** 2019-02-01

**Authors:** Mukharram M. Bikbov, Gyulli M. Kazakbaeva, Timur R. Gilmanshin, Rinat M. Zainullin, Inga I. Arslangareeva, Venera F. Salavatova, Guzel M. Bikbova, Songhomitra Panda-Jonas, Nikolai A. Nikitin, Artur F. Zaynetdinov, Ildar F. Nuriev, Renat I. Khikmatullin, Yulia V. Uzianbaeva, Dilya F. Yakupova, Said K. Aminev, Jost B. Jonas

**Affiliations:** 1 Ufa Eye Research Institute, Ufa, Bashkortostan, Russia; 2 Augenpraxis Prof. Jonas, Seegartenklinik, Heidelberg, Germany; 3 Department of Ophthalmology, Medical Faculty Mannheim of the Ruprecht-Karls-University of Heidelberg, Mannheim, Germany; Federal University of São Paulo, BRAZIL

## Abstract

**Purpose:**

To assess the normal distribution of axial length and its associations in a population of Russia.

**Methods:**

The population-based Ural Eye and Medical Study included 5,899 (80.5%) individuals out of 7328 eligible individuals aged 40+ years. The participants underwent an ocular and systemic examination. Axial length was measured sonographically (Ultra-compact A/B/P ultrasound system, Quantel Medical, Cournon d'Auvergne, France).

**Results:**

Biometric data were available for 5707 (96.7%) individuals with a mean age of 58.8±10.6 years (range:40–94 years; 25%, 50%, 75% quartile: 51.0, 58.0, 66.0 years, respectively). Mean axial length was 23.30±1.10 mm (range: 19.02–32.87mm; 95% confidence interval (CI): 21.36–25.89; 25%, 50%, 75% quartile: 22.65mm, 23.23mm, 23.88mm, resp.). Prevalences of moderate myopia (axial length:24.5-<26.5mm) and high myopia (axial length >26.5mm) were 555/5707 (8.7%;95%CI:9.0,10.5) and 78/5707 (1.4%;95%CI:1.1,1.7), respectively. Longer axial length (mean:23.30±1.10mm) was associated (correlation coefficient r^2^:0.70) with older age (*P*<0.001;standardized regression coefficient beta:0.14), taller body height (*P*<0.001;beta:0.07), higher level of education (*P*<0.001;beta:0.04), higher intraocular pressure (*P*<0.001;beta:0.03), more myopic spherical refractive error (*P*<0.001;beta:-0.55), lower corneal refractive power (*P*<0.001;beta:-0.44), deeper anterior chamber depth (*P*<0.001;beta:0.20), wider anterior chamber angle (*P*<0.001;beta:0.05), thinner peripapillary retinal nerve fiber layer thickness (*P*<0.001;beta:-0.04), higher degree of macular fundus tessellation (*P*<0.001;beta:0.08), lower prevalence of epiretinal membranes (*P* = 0.01;beta-0.02) and pseudoexfoliation (*P* = 0.007;beta:-0.02) and higher prevalence of myopic maculopathy (*P*<0.001;beta:0.08). In that model, prevalence of age-related macular degeneration (any type: *P* = 0.84; early type: *P* = 0.46), diabetic retinopathy (*P* = 0.16), and region of habitation (*P* = 0.27) were not significantly associated with axial length.

**Conclusions:**

Mean axial length in this typically multi-ethnic Russian study population was comparable with values from populations in Singapore and Beijing. In contrast to previous studies, axial length was not significantly related with the prevalences of age-related macular degeneration and diabetic retinopathy or region of habitation.

## Introduction

Myopia and hyperopia belong to the most common health disorders worldwide [1,2]. For the year 2000, 1,406 million people (22.9% of the world population) have been estimated to be myopic and 163 million people (2.7% of the world population) to have high myopia [2]. These figures were predicted to rise by the year 2050 to 4,758 million people (49.8% of the world population) with myopia and 938 million people (9.8% of the world population) with high myopia [2]. Since high myopia can lead to myopic maculopathy and optic neuropathy, myopia has been estimated to become the most common cause for irreversible blindness worldwide [1–5]. In particular the association between high myopia and glaucomatous optic neuropathy is of importance since in a recent hospital-based study the prevalence of glaucomatous optic neuropathy markedly increased with axial length beyond an axial length of 26.5 mm, from 12.2% in eyes with an axial length of <26.5mm to 28.5% in eyes with an axial length of ≥26.5mm, to 32.6% in eyes with an axial length of ≥28mm, to 36.0% in eyes with an axial length of ≥29mm, and to 42.1% in eyes with an axial length of ≥30mm [6]. These observations confirmed findings made in previous studies [7–13]. In addition, hyperopia is a major risk factor for strabismus and amblyopia, angle-closure glaucoma, age-related macular degeneration and diabetic retinopathy, all of which are also common vision-threatening diseases [1,14,15]. It is therefore important to know the prevalence of refractive errors in the general population. Although data on the prevalence of refractive errors or of the distribution of ocular axial length as the surrogate of refractive error have been assessed in previous investigations performed in many countries and including the Barbados Eye Study, Blue Mountains Eye Study, Tanjong Pagar Study, Beijing Eye Study and Los Angeles Latino Eye Study Group, information about the distribution of ocular axial length in Russia as the largest and one of the most populous countries worldwide has been scarce so far [2, 14–21]. We therefore conducted this population-based study to assess the distribution of axial length and its associations in a typically multi-ethnic population in Russia. To reduce the risk of a confounding effect by a causative factor which would not have been included into the statistical analysis, we correlated the main outcome parameter, i.e. axial length, with a multitude of ocular and systemic parameters. These factors included parameters describing the socioeconomic background (e.g., ownership of a car or computer), parameters quantifying the amount of physical activity, anthropomorphic variables and prevalences of systemic diseases such as diabetes mellitus, to mention only a few.

## Methods

The Ural Eye and Medical Study (UEMS) is a population-based investigation which was conducted in the republic of Bashkortostan / Russia [22,23]. The study design was approved by the Ethics Committee of the Academic Council of the Ufa Eye Research Institute. Informed written consent was obtained from all participants. The republic of Bashkortostan with a population of about 4.07 million people is located at the south-western end of the Ural Mountains. Its capital Ufa is situated about 1300 km east of Moscow. Its population of 1.1 million inhabitants includes Russians, Bashkirs, Tatars, Ukrainians and other ethnicities [24]. The study regions were a rural area in the Karmaskalinsky District in a distance of 65 km from Ufa, and the urban area of Kirovskii in the city of Ufa. Living in the study regions and an age of 40+ years were the inclusion criterion for the study.

The series of examination started with a standardized interview conducted by trained social workers and consisting of more than 250 questions on socioeconomic parameters, diet, smoking habits or other types of tobacco consumption, daily physical activity, alcohol consumption, depression, and known diagnosis and therapy of major diseases such as diabetes mellitus and arterial hypertension. The data were collected using the Guidelines for Accurate and Transparent Health Estimates Reporting (GATHER statement guidelines) [25].

We measured the anthropomorphic parameters of body height, body weight and circumference of the hip and waist, arterial blood pressure and pulse rate. For the measurement of body height, we used a stadiometer, the shoes of the study participants were removed and the study participants were asked to stand upright as much as possible and with the head raised upright. The body weight was determined by a weighing machine, with the shoes and heavy clothes such as coats and pullovers removed. The handgrip strength was determined using a dynamometer (dynamometer—dk 140, ZAO Nizhnetagilskiy Medical Instrument Plant, Nizhniy Tagil, Russia). Blood samples taken under fasting conditions were biochemically examined including the determination of the serum concentrations of glucose, blood lipids, C-reactive protein, erythrocyte sedimentation rate, hemoglobin and other substances. The pulmonary function was assessed by spirometry (Riester spirotest, Riester Company, Jungingen, Germany). Arterial hypertension was defined by a systolic blood pressure ≥140 mmHg and/or a diastolic blood pressure ≥90 mmHg, and/or self-reported history or current treatment of arterial hypertension with antihypertensive medication. Diabetes mellitus was defined by a glucose concentration ≥7.0 mmol/L or a self-reported history of physician diagnosis of diabetes mellitus or a history of drug treatment for diabetes (insulin or oral hypoglycemic agents). Depression was assessed applying the Center for Epidemiologic Studies Depression Scale (CES-D) Scoresheet. The study design has been described in detail recently [22,23].

The series of ophthalmologic examination consisted of determination of best corrected visual acuity by automated refractometry (Auto-Ref/Keratometer HRK-7000A HUVITZ Co, Ltd., Gyeonggi-do, Korea) and subjective refractometry, imaging of the anterior ocular segment (Pentacam HR, Typ70900, OCULUS, Optikgeräte GmbH Co., Wetzlar, Germany), slit lamp biomicroscopy of the anterior and posterior ocular segment, non-contact tonometry (Tonometer Kowa KT-800, Kowa Company Ltd., Hamamatsu City, Japan), assessment of the presence of pseudoexfoliation of the lens after medical mydriasis, photography of the cornea and lens (Topcon slit lamp and camera, Topcon Corp. Tokyo, Japan), and of the optic disc and macula (VISUCAM 500, Carl Zeiss Meditec AG, Jena, Germany), spectral-domain optical coherence tomography (RS-3000, NIDEK co., Ltd., Aichi Japan) of the optic nerve head and macula, and assessment of axial length by sonography (Ultra-compact A/B/P ultrasound system, Compact touch; Quantel Medical, Cournon d'Auvergne, France).

Only one randomly selected eye per study participant was included into the statistical analysis. Using a statistical software package (SPSS for Windows, version 25.0, SPSS, Chicago, IL), we first assessed the mean values ± standard deviations of the main outcome parameters and conducted univariate analyses of the associations between axial length and other ocular and systemic parameters. We then performed multivariate regression analyses with axial length as the dependent parameter and as independent parameters all those variables that were associated significantly with axial length in the univariate analyses. In a step-by-step manner, we dropped those variables out of the list of independent parameters that either showed a high collinearity or that were no longer significantly associated with axial length. We assessed the standardized regression coefficient beta, the non-standardized regression coefficient B and its 95% confidence intervals (CI). All *P*-values were two-sided and considered statistically significant when the values were less than 0.05.

## Results

Out of 7328 subjects eligible for inclusion into the UEMS, the UEMS included 5899 (80.5%) participants (2580 (43.7%) men). Available information on axial length was obtained for 5707 (or 96.7% of the total UEMS study population) individuals, while for the remaining 192 (3.3%) individuals a reliable biometry of the axial length could not be performed due to reasons such as cataract and insufficient cooperation of the study participant. The group of participants in the present study as compared with the group of non-participants was significantly (*P*<0.001) younger (mean age: 58.8 ± 10.6 years; range: 40–94 years), was living more often (*P*<0.001) in the urban region and had a higher (*P*<0.001) level of education (Table 1). Both groups did not differ significantly in sex (*P* = 0.42) and anthropomorphic parameters (all *P*>0.15) (Table 1). The gender and age distribution in the study population corresponded to the gender and age distribution in the Russian population according to the most recent census carried out in 2010 [26].

**Table 1:** Demographic parameters of participants of the present study and the non-participants.

Parameter	Study Participants	Non-Participants	*P*-Value
n	5707	192	
Age (Years)	58.8 ± 10.6 (range: 40–94 years)	63.3 ± 11.2	<0.001
Sex (Men / Women)	2502 / 3205 (43.8% / 56.2%)	78 / 114 (40.6% / 59.4%)	0.42
Region of Habitation (Urban / Rural)	2342 / 3365 (41.0% / 59.0%)	157 / 35 (81.8% / 18.2%)	<0.001
Body Height (cm)	164.8 ± 8.8	164.2 ± 8.9	0.36
Body Weight (kg)	75.9 ± 14.6	74.4 ± 14.6	0.16
Body Mass Index (kg/m^2^)	27.9 ± 5.0	27.6 ±5.2	0.40
Hip Circumference (cm)	103.6 ± 12.6	103.7 ± 11.5	0.98
Waist Circumference (cm)	94.0 ± 13.3	93.6 ± 13.1	0.68
Waist / Hip Circumference Ratio	0.91 ± 0.09	0.90 ± 0.07	0.33
Level of Education (Illiteracy / Passing 5^th^ Grade / 8th Grade / 10th Grade / 11th Grade / Graduates / Post Graduates / Specialized Secondary Education.	16 (0.3%) / 102 (1.8%) / 564 (9.9%) / 643 (11.3%) / 747 (13.1%) / 1966 (34.4%) / 51 (0.9%) / 1617 (28.3%)	1 (0.5%) / 2 (1.0%) / 29 (15.1%) / 16 (8.3%) / 35 (18.2%) / 86 (44.8%) / 1 (0.5%) / 21 (10.9%)	<0.001

**Fig 1:**
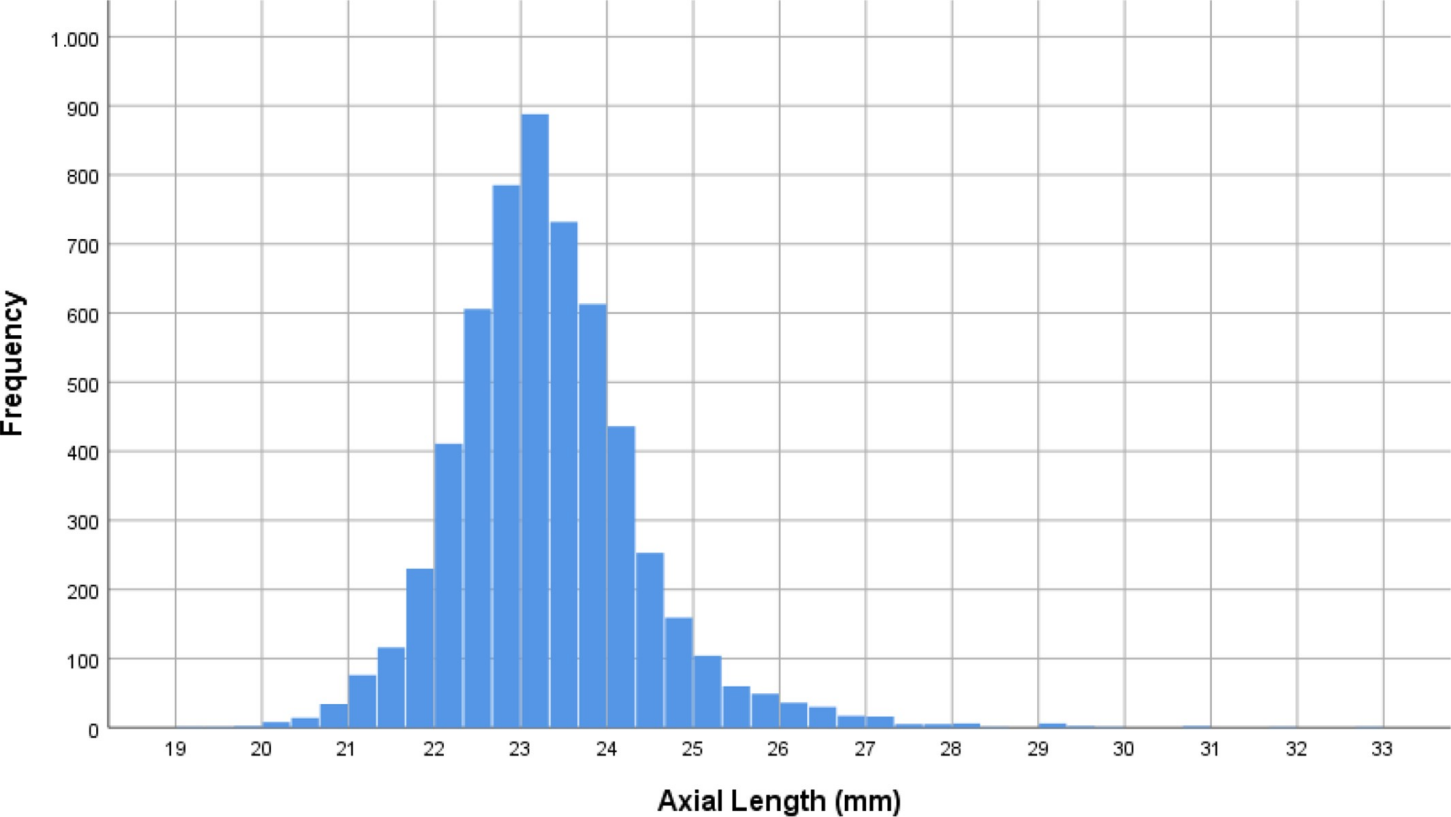
Histogram showing the distribution of axial length in the Ural Eye and Medical Study.

**Fig 2:**
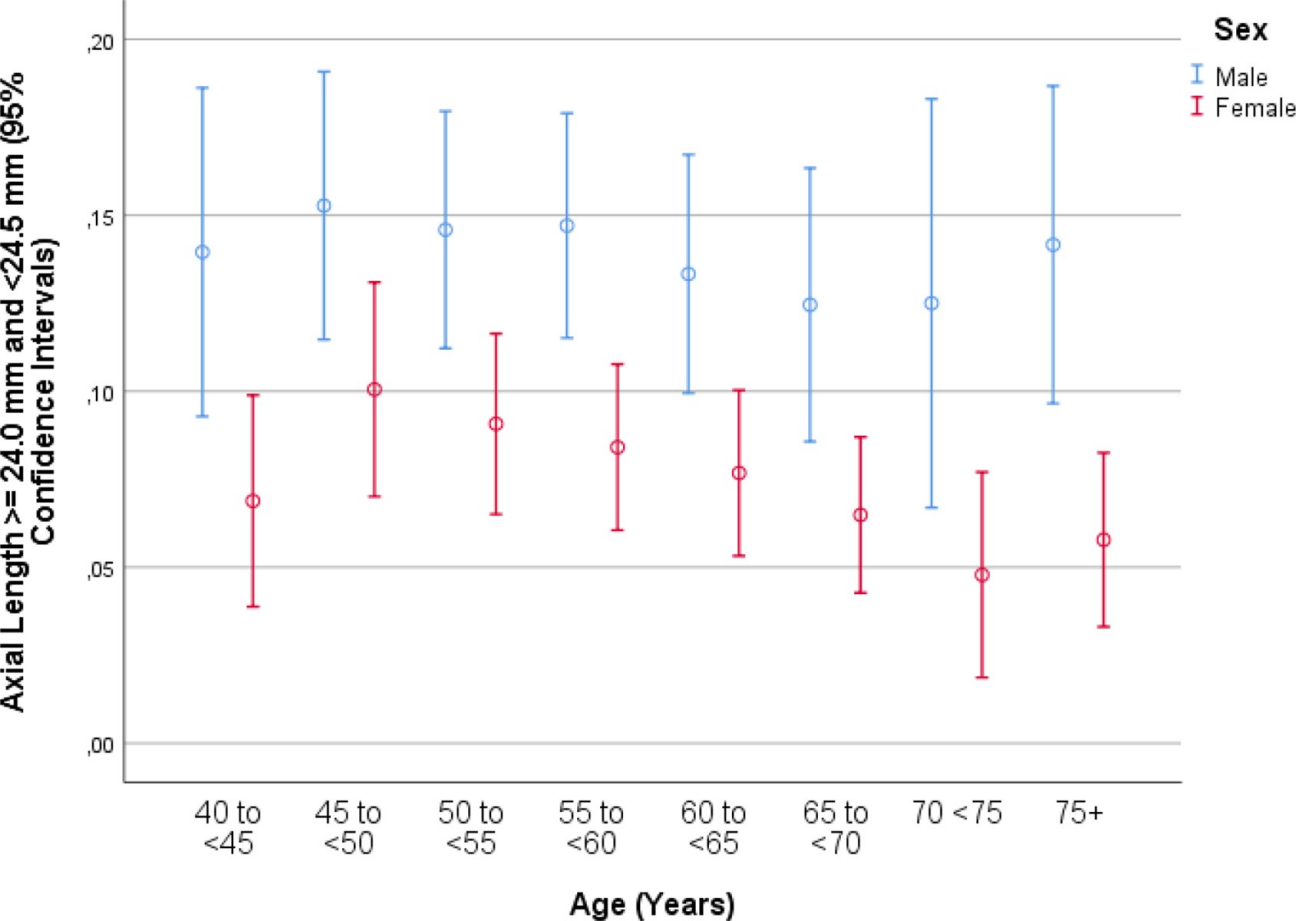
Graph showing the prevalence of minor axial myopia stratified by age and gender in the Ural Eye and Medical Study.

**Fig 3:**
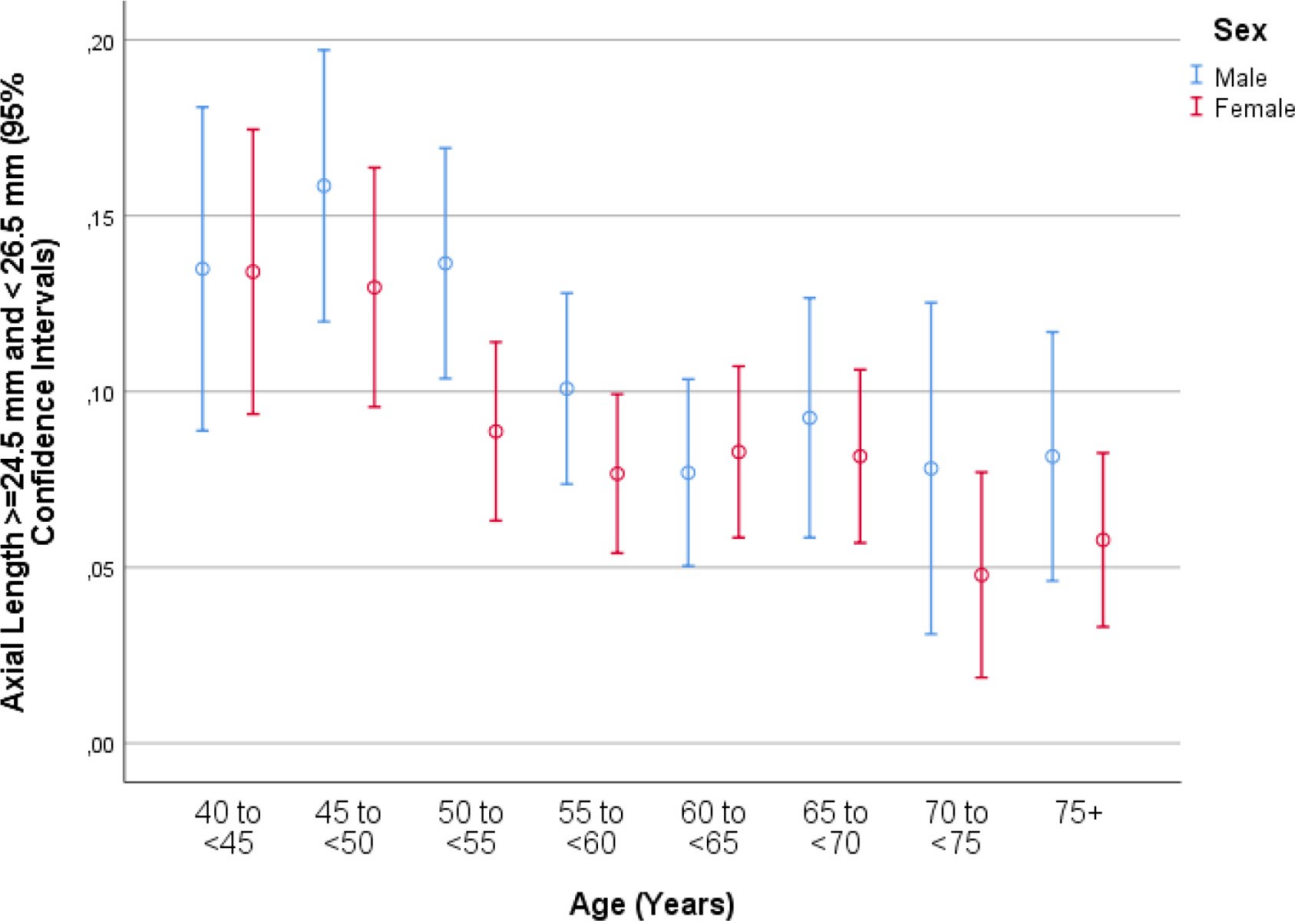
Graph showing the prevalence of moderate axial myopia stratified by age and gender in the Ural Eye and Medical Study.

**Fig 4:**
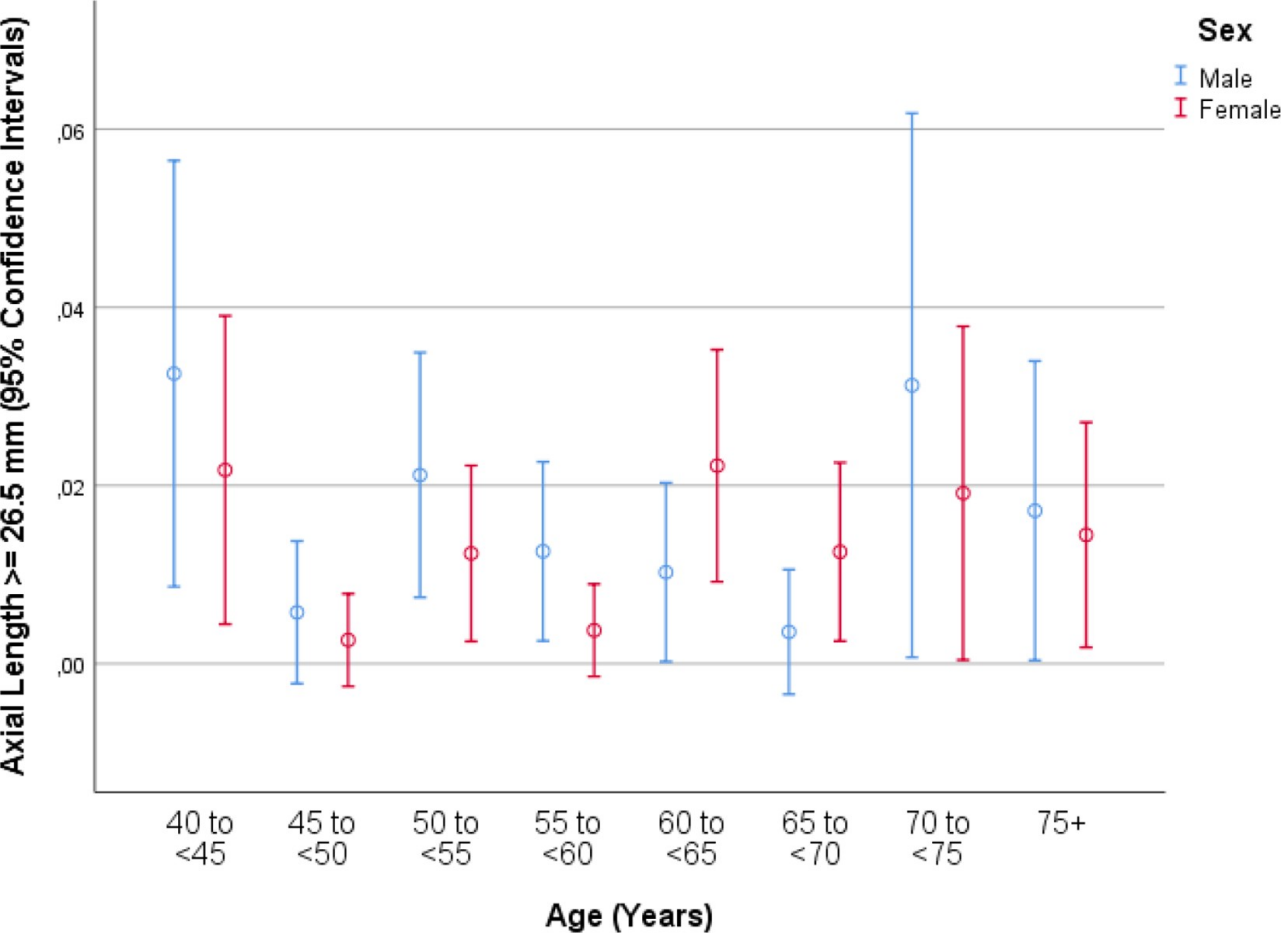
Graph showing the prevalence of high axial myopia stratified by age and gender in the Ural Eye and Medical Study.

Mean axial length was 23.30 ± 1.10 mm (median: 23.21 mm; range: 19.02 mm to 32.87 mm) (Fig 1). Mean axial length was 23.32 ± 1.10 mm (median: 23.23 mm; range: 19.78–32.87mm) in the right eyes and 23.29 ± 1.09 mm (median: 23.19mm; range: 19.02–32.20mm) in the left eyes. The prevalences of any myopia (defined by axial length ≥24.0mm), minor myopia (axial length: 24.0 to <24.50mm) (Fig 2), moderate myopia (axial length: 24.5 to <26.5mm) (Fig 3) and high myopia (axial length >26.5mm) (Fig 4)were 1229/5707 (21.5%; 95%CI: 20.5, 22.6), 596/5707 (10.4%; 95%CI: 9.7, 11.3), 555/5707 (8.7%; 95%CI: 9.0, 10.5) and 78/5707 (1.4%; 95%CI: 1.1, 1.7), respectively.

**Fig 5:**
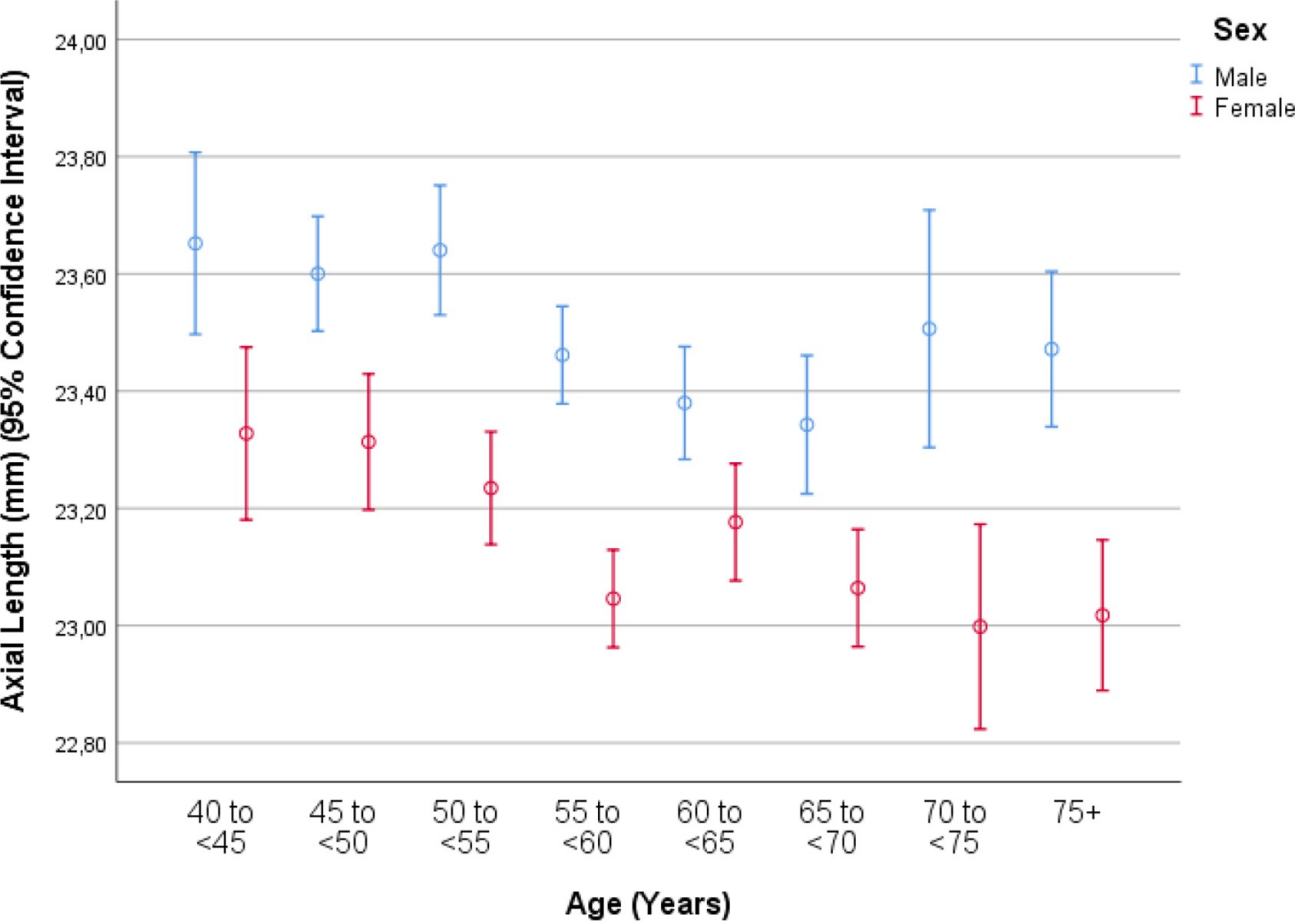
Graph showing the distribution of axial length in the Ural Eye and Medical Study, stratified by age and sex.

In univariate analyses, axial length was associated with the systemic parameters of younger age (*P*<0.001) (Fig 5), male sex (*P*<0.001) (Fig 2), urban region of habitation (*P* = 0.001), married family status (*P*<0.001), greater body height (*P*<0.001), larger body weight (*P*<0.001) and lower body mass index (*P* = 0.04), higher level of education (*P*<0.001) and higher frequency of ownership of a house (*P* = 0.003) and second house (*P* = 0.002), smartphone (*P* = 0.003), car (*P*<0.001), two-wheeler (*P*<0.001) and computer (*P*<0.001), longer working days (*P*<0.001) and more sitting at work (*P* = 0.02), higher frequency (*P* = 0.02) of, more days with (*P*<0.001) and more hours per day with (*P*<0.001) vigorous physical activity at work, higher frequency of walking or bicycling (*P* = 0.01), lower prevalence of a positive history of arterial hypertension (*P*<0.001), arthritis (*P*<0.001), neck pain (*P* = 0.02) and headache (*P*<0.001), diabetes mellitus (*P* = 0.006), thyreopathy (*P*<0.001) and menopause (*P*<0.001), higher blood concentrations of bilirubin (*P*<0.001), creatinine (*P* = 0.02), total protein (*P* = 0.03) and hemoglobin (*P*<0.001), lower blood concentrations of high-density lipoproteins (*P* = 0.001), glucose (*P* = 0.009) and urea (*P* = 0.02), shorter prothrombin time (*P* = 0.01) and higher INR (international normalized ratio) value (*P* = 0.03), higher count of erythrocytes (*P*<0.001), higher percentage of segment nuclear granulocytes (*P* = 0.001) and lower percentage of eosinophilic granulocytes (*P* = 0.02) and lymphocytes 02), lower erythrocyte sedimentation rate (*P*<0.001), lower prevalence of diabetes mellitus (*P* = 0.005), lower systolic blood pressure (*P*<0.001), higher number of days per week with intake consumption of vegetables (*P*<0.001), higher prevalence of alcohol consumption (*P*<0.001), lower depression score (*P*<0.001) and anxiety score (*P*<0.001), and higher dynamometric force in the hands (*P*<0.001) (Table 2). Longer axial length was correlated with the ocular parameters of a worse best corrected visual acuity (*P*<0.001), higher intraocular pressure (*P* = 0.008), more marked cylindrical refractive error (*P*<0.001) and more marked spherical refractive error (*P*<0.001), lower corneal refractive power (*P*<0.001), thicker central corneal thickness (*P* = 0.002), lower corneal volume, larger anterior chamber depth (*P*<0.001), higher anterior chamber volume (*P*<0.001), wider anterior chamber angle (*P*<0.001), thinner lens (*P*<0.001), lower area (*P*<0.001) and volume (*P*<0.001) of macular pigment, higher retinal thickness 300 μm temporal to the fovea, thinner peripapillary retinal nerve fiber layer thickness (*P*<0.001), lower degree of nuclear cataract (*P* = 0.001), lower prevalence of any stage (*P*<0.001) and of the early stage (*P* = 0.001) of age-related macular degeneration, higher prevalence of myopic maculopathy (*P*<0.001), higher degree of fundus tessellation in the macular region (*P*<0.001) and the peripapillary region (*P*<0.001), higher frequency of epiretinal membrane (*P* = 0.006), and pseudoexfoliation syndrome (*P* = 0.04) (Table 3).

**Table 2:** Associations (univariate analysis) between axial length score and systemic parameters in the Ural Eye and Medical Study.

Parameter	*P*-Value		Standardized Regression Coefficient beta	Non-Standardized Regression Coefficient B	95% Confidence Interval of B
Age (Years)	<0.001		-0.09	-0.01	-0.01, -0.01
Gender: Women / Men	<0.001		-0.16	-0.36	-0.42, -0.30
Urban / rural region of habitation	0.001		-0.04	-0.10	-0.15, -0.04
Family status: Married versus any other status	<0.001		0.05	0.13	0.07, 0.20
Family type: Joint (three generations) / nuclear (two generations) / single / family of 2 people	0.03		0.03	0.03	0.003, 0.05
Ethnicity: Russian / any other ethnicity	0.053		0.03	0.07	-0.001, 0.14
Body height (cm)	<0.001		0.22	0.03	0.02, 0.03
Body weight (kg)	<0.001		0.10	0.007	0.005, 0.009
Body mass index (kg/m^2^)	0.04		-0.03	-0.006	-0.12, 0.000
Waist circumference (cm)	0.80				
Hip circumference cm)	0.55				
Waist-Hip-Ratio	0.29				
Socioeconomic parameters					
Level of education	<0.001		0.11	0.07	0.05, 0.09
Monthly Income (Below poverty line / average / above average / high)	0.53				
Own ownership of house (yes / no)	0.003		-0.04	-0.11	-0.18, -0.04
Own ownership of refrigerator (yes / no)	0.30				
Own ownership of second house (yes / no)	0.002		0.06	0.16	0.06, 0.25
Own ownership of telephone (yes / no)	0.79				
Own ownership of smartphone (yes / no)	0.003		0.05	0.11	0.04, 0.19
Own ownership of television set (yes / no)	0.33				
Own ownership of car (yes / no)	<0.001		0.08	0.18	0.10, 0.26
Own ownership of two-wheeler (yes / no)	<0.001		0.05	0.11	0.05, 0.17
Own ownership of tractor (yes / no)	0.35				
Own ownership of bullock cart (yes / no)	0.71				
Own ownership of computer / laptop (yes / no)	<0.001		0.10	0.23	0.16, 0.31
Physical activity					
How long is your usual work day? (Minutes)	<0.001		0.05	0.00	0.00, 0.00
Does your work involve mostly sitting or standing with less than 10 minutes of walking at a time? (Yes / No)	0.02		0.03	0.08	0.01, 0.14
Does your work involve physically vigorous activity (like heavy lifting or digging) or physically moderate intensity activity (like brisk walking or carrying light loads) (Yes / No)	0.02		-0.03	-0.07	-0.14, -0.01
How many days a week do you do such physically vigorous activity during work? (Yes / No)	0.001		-0.06	-0.04	-0.07, -0.02
On a usual day how much time do you spend on such physically vigorous work during work? (Minutes)	<0.001		0.05	0.00	0.00, 0.00
Does your work involve physically moderate-intensive activity, like brisk walking or carrying light loads for at least 10 minutes at a time?	0.84				
Do you walk or use a bicycle (pedal cycle) for at least 10 minutes continuously to get to and from places?	0.01		0.03	0.12	0.03, 0.21
In your leisure time, do you do any moderate intensity activities like brisk walking, cycling or swimming for at least 10 minutes at a time?	0.43				
Over the past 7 days, how much time did you spend sitting or reclining on a typical day?	0.52				
History of diseases					
History of angina pectoris	0.91				
History of asthma	0.88				
History of arterial hypertension	<0.001		-0.05	-0.11	-0.17, -0.06
History of arthritis	<0.001		-0.07	-0.17	-0.23, -0.10
History of previous bone fractures	0.43				
History of low back pain	0.85				
History of thoracic spine pain	0.10		-0.02	-0.06	-0.13, 0.01
History of neck pain	0.02		-0.03	-0.08	-0.14, -0.01
History of headache	<0.001		-0.07	-0.16	-0.22, -0.10
History of cancer	0.20				
History of cardiovascular disorders including stroke	0.35				
History of dementia	0.36				
History of diabetes mellitus	0.006		-0.04	-0.15	-0.25, -0.04
History of diarrhea	0.83				
History of iron-deficiency anemia	0.68				
History of low blood pressure and hospital admittance	0.59				
History of osteoarthritis	0.07		-0.03	-0.07	-0.15, 0.01
History of skin disease	0.32				
History of thyreopathy	<0.001		-0.05	-0.19	-0.29, -0.10
History of tumbling	0.17		-0.02	-0.05	-0.13, 0.02
History of unconsciousness	0.47				
Age of the last menstrual bleeding (years)	0.83				
Age of last regular menstrual bleeding (years)	0.73				
History of menopause	<0.001		-0.07	-0.19	-0.30, -0.09
Blood concentrations (mmol/L) of:					
Alanine aminotransferase (IU/L)	0.27				
Aspartate aminotransferase (IU/L)	0.06		0.03	0.003	0.000, 0.005
Bilirubin, total (μmol/L)	<0.001		0.06	0.006	0.003, 0.008
High-density lipoproteins (mmol/L)	0.001		-0.05	-0.06	-0.09, -0.03
Low-density lipoproteins (mmol/L)	0.35				
Cholesterol (mmol/L)	0.07		-0.02	-0.02	-0.03, 0.001
Triglycerides (mmol/L)	0.25				
Rheumatoid factor (IU/mL)	0.45				
Erythrocyte sedimentation rate (mm / hour)	<0.001		-0.06	-0.006	-0.008, -0.003
Glucose (mmol/L)	0.009		-0.04	-0.02	-0.04, -0.01
Prevalence of diabetes mellitus	0.005		-0.04	-0.13	-0.22, -0.04
Creatinine (μmol/L)	0.02		0.03	0.001	0.000, 0.003
Urea (mmol/L)	0.02		-0.03	-0.02	-0.04, -0.004
Residual nitrogen (g/L)	0.11		-0.02	-0.32	-0.71, 0.08
Total protein (g/L)	0.03		0.03	0.005	0.001, 0.01
International normalized ratio (INR)	0.03		0.03	0.23	0.3, 0.43
Prothrombin time (%)	0.01		-0.03	-0.004	-0.006, -0.001
Hemoglobin	<0.001		0.06	0.005	0.003, 0.007
Erythrocytes (10^6^ cells / μL)	<0.001		0.07	0.19	0.11, 0.26
Leukocytes (10^9^ cells / L)	0.27				
Rod-core granulocyte (% of leukocytes)	0.37				
Segment nuclear granulocyte (% of leukocytes)	0.001		0.05	0.007	0.003, 0.010
Eosinophil granulocytes (% of leukocytes)	0.02		-0.04	-0.03	-0.06, -0.01
Lymphocytes (% of leukocytes)	0.02		-0.03	-0.005	-0.01, -0.001
Monocytes (% of leukocytes)	0.14		-0.02	-0.009	-0.022, 0.003
Blood pressure, systolic (mmHg)	<0.001		-0.05	-0.003	-0.004, -0.001
Blood pressure, diastolic (mmHg)	0.57				
Blood pressure, mean (mmHg)	0.28				
Prevalence of arterial hypertension	0.12		-0.02	-0.05	-0.10, 0.01
Prevalence of chronic obstructive pulmonary disease	0.64				
Diet					
Vegetarian diet / mixed diet	0.94				
Number of meals per day	0.26				
In a week how many days do you eat fruits?	0.15		0.02	0.01	-0.004, 0.03
In a week how many days do you eat vegetables?	<0.001		0.05	0.001	0.000, 0.001
Type of oil used for cooking: vegetable oil / non-vegetable oil	0.39				
Salt consumed per day (g)	0.77				
Degree of processing of meat (weak / medium / well done)	0.44				
Smoking					
Do you currently smoke any tobacco products? (yes)	0.10		0.02	0.07	-0.02, 0.16
Do you smoke daily? (yes / no)	0.15		0.02	0.07	-0.02, 0.15
Package years (package = 20 cigarettes)	0.50				
Alcohol Consumption					
Alcohol consumed such as beer, whisky, rum, gin brandy or other local products? (yes / no)	<0.001		0.06	0.15	0.08, 0.22
How many alcoholic drinks do you have on a typical day when you are drinking)	0.43				
How often do you have 6 or more drinks on one occasion? (never / rarely / sometimes / often / cannot say)	0.08		-0.05	-0.07	-0.14, 0.01
Depression					
Depression score		<0.001	-0.07	-0.02	-0.03, -0.01
State-Trait Anxiety Inventory (STAI)					
Anxiety score		<0.001	-0.06	-0.02	-0.03, -0.01
Dynamometry					
Manual dynamometry, right hand (dekaNewton)		<0.001	0.18	0.02	0.02, 0.02
Manual dynamometry, left hand (dekaNewton)		<0.001	0.18	0.02	0.02, 0.02

**Table 3:** Associations (univariate analysis) between axial length score and ophthalmological parameters in the Ural Eye and Medical Study.

Parameter	*P*-Value	Standardized Regression Coefficient beta	Non-Standardized Regression Coefficient B	95% Confidence Interval of B
Best Corrected Visual Acuity (logMAR)	<0.001	0.06	0.33	0.16, 0.50
Intraocular Pressure (mmHg)	0.008	0.04	0.01	0.003, 0.02
Refractive Error, Spherical Value (Diopters)	<0.001	-0.59	-0.29	-0.30, -0.28
Refractive Error, Cylindrical Value (Diopters)	<0.001	-0.11	-0.15	-0.18, -0.11
Refractive Error, Spherical Equivalent (Diopters)	<0.001	-0.59	-0.28	0.29, -0.27
Corneal Refractive Power (Diopters)	<0.001	-0.41	-0.26	-0.28, -0.25
Central Corneal Thickness (μm)	0.002	0.04	0.001	0.001, 0.002
Corneal Volume	<0.001	-0.09	-0.02	-0.03, -0.02
Anterior Chamber Depth (mm)	<0.001	0.42	0.96	0.90, 1.01
Anterior Chamber Volume	<0.001	0.54	0.02	0.02, 0.02
Anterior Chamber Angle	<0.001	0.32	0.05	0.05, 0.05
Lens Thickness (mm)	<0.001	-0.10	-0.26	-0.33, -0.19
Macular Pigment Density Area	<0.001	-0.08	-0.000005	0.000, 0.000
Macular Pigment Density Volume	<0.001	-0.09	-0.00005	0.000, 0.000
Retinal Thickness (Total), Fovea	0.61			
Retinal Thickness (Total), 300 μm Temporal to the Fovea	0.01	0.04	0.001	0.000, 0.002
Retinal Thickness (Total), 300 μm Nasal to the Fovea	0.08	0.02	0.001	0.000, 0.001
Retinal Nerve Fiber Layer Thickness (μm)	<0.001	-0.18	-0.009	-0.010, -0.007
Nuclear Cataract	0.001	-0.05	-0.05	-0.08, -0.02
Cortical Cataract	0.14			
Subcapsular Cataract	0.31			
Age-Related Macular Degeneration, Any	<0.001	-0.06	-0.19	-0.29, -0.09
Age-Related Macular Degeneration, Early Stage	0.001	-0.05	-0.21	-0.32, -0.09
Age-Related Macular Degeneration, Intermediate Stage	0.07	-0.03	-0.18	-0.38, 0.02
Age-Related Macular Degeneration, Late Stage	0.83			
Myopic Maculopathy	<0.001	0.43	1.22	1.15, 1.28
Diabetic Retinopathy	0.26			
Retinitis Pigmentosa	0.23			
Polypoidal Choroidal Vasculopathy	0.99			
Central Serous Choroidopathy	0.52			
Fundus Tessellation, Macula Region	<0.001	0.31	0.40	0.36, 0.43
Fundus Tessellation, Peripapillary Region	<0.001	0.32	0.36	0.33, 0.39
Epiretinal Membranes	0.006	0.04	0.22	0.06, 0.38
Macular Holes, Full Thickness	0.41			
Prevalence of Pseudoexfoliation	0.04	0.03	0.004	0.000, 0.008

In a next step of the statistical analysis, we performed a multivariate linear regression analysis with axial length as the dependent parameter and, as independent parameters, all variables that were significantly associated with axial length in the univariate analysis (Tables 2 and 3). Due to high collinearity, we removed step by step the parameters of body weight (variance inflation factor (VIF): 184), dynamometry of the left hand (VIF: 8.0), INR ratio value (VIF: 7.5), hemoglobin (VIF: 10.0), percentage of segment nuclear leukocytes (VIF: 5.9), history of diabetes (VIF: 5.0), time spent with vigorous activity per day (VIF: 3.8), and anterior chamber volume (VIF: 9.2). Due to a lack of statistical significance, we dropped the parameters of vigorous activity during work (*P* = 0.37), percentage of eosinophilic leukocytes (*P* = 0.68), work with a major part of sitting or standing (*P* = 0.86), dynamometry of the right hand (*P* = 0.99), ethnicity (Russian versus non-Russian) (*P* = 0.92), ownership of a car (*P* = 0.86), laptop (*P* = 0.73), use of a bicycle (*P* = 0.62), history of neck pain (*P* = 0.95) and headache (*P* = 0.68), blood concentration of bilirubin (*P* = 0.77) and creatinine (*P* = 0.95), erythrocyte count (*P* = 0.85), erythrocyte sedimentation rate (*P* = 0.80), anxiety score (*P* = 0.68), history of osteoarthritis (*P* = 0.55), blood concentration of high-density lipoproteins (*P* = 0.85) and aspartate aminotransferase (*P* = 0.45), history of arthritis (*P* = 0.67), family status (*P* = 0.64), region of habitation (*P* = 0.47), ownership of house (*P* = 0.38), alcohol consumption of more than 6 drinks per occasion (*P* = 0.22), gender (*P* = 0.86), thoracic spine pain (*P* = 0.93), number of days with intake of vegetables (*P* = 0.76), lymphocyte count (*P* = 0.50), blood concentration of glucose (*P* = 0.41), any alcohol consumption (*P* = 0.65), macular pigment density area (*P* = 0.87), history of thyreopathy (*P* = 0.60), length of usual working day (*P* = 0.28), blood concentration of total protein (*P* = 0.51), lens thickness (*P* = 0.85), retinal thickness 300 μm nasal to the fovea (*P* = 0.72), peripapillary fundus tessellation (VIF: 2.5), blood concentration of urea (*P* = 0.25), systolic blood pressure (*P* = 0.97), body mass index (*P* = 0.73), best corrected visual acuity (*P* = 0.95), current smoking (*P* = 0.31), degree of nuclear cataract (*P* = 0.19), prevalence of diabetes mellitus (*P* = 0.23), prothrombin index (*P* = 0.12), central corneal thickness (*P* = 0.42), retinal thickness 300 μm temporal to the fovea (*P* = 0.37), blood concentration of cholesterol (*P* = 0.10), cylindrical refractive error (*P* = 0.16), spherical equivalent of the refractive error (*P* = 0.16), depression score (*P* = 0.10), and prevalence of early age-related macular degeneration (*P* = 0.82).

In the final model, longer axial length was associated (correlation coefficient r^2^: 0.70) with the systemic parameters of older age (*P*<0.001), taller body height (*P*<0.001), higher prevalence of arterial hypertension (*P* = 0.02), and higher level of education (*P*<0.001); and with the ocular parameters of a higher intraocular pressure (*P*<0.001), more myopic spherical refractive error (*P*<0.001), lower corneal refractive power (*P*<0.001), lower corneal volume (*P*<0.001), deeper anterior chamber depth (*P*<0.001) and wider anterior chamber angle (*P*<0.001), lower macular density volume (*P*<0.001), thinner peripapillary retinal nerve fiber layer thickness (*P*<0.001), higher degree of macular fundus tessellation (*P*<0.001), higher prevalence of myopic maculopathy (*P*<0.001), lower prevalence of epiretinal membranes (*P* = 0.01), and lower prevalence of pseudoexfoliation (*P* = 0.007) (Table 4). If the prevalence of age-related macular degeneration (any type: *P* = 0.84; early type: *P* = 0.46), the prevalence of diabetic retinopathy (*P* = 0.16) or the region of habitation (*P* = 0.27) were added to the model, these parameters were not significantly associated with axial length. If highly myopic eyes (defined as an axial length of longer than 26.5 mm) were excluded, almost identical results were obtained. Within the highly myopic group (n = 79), the associations of the final model were not statistically significant. When ownership of a two-wheeler (*P* = 0.06), a second house (*P* = 0.02) and smartphone (*P* = 0.02) were replaced by the level of education, the latter was significantly correlated with axial length.

**Table 4:** Associations (multivariate analysis) between axial length and systemic and ocular parameters in the Ural Eye and Medical Study.

	*P*-Value	Standardized Coefficient Beta	Non-Standardized Regression Coefficient B	95% Confidence Intervals of B	Variance Inflation Factor
Age (Years)	<0.001	0.14	0.02	0.01, 0.02	1.79
Body Height (cm)	<0.001	0.07	0.008	0.006, 0.01	1.14
Arterial Hypertension	0.02	0.02	0.05	0.009, 0.09	1.11
Level of Education	<0.001	0.04	0.03	0.04, 0.92	1.09
Intraocular Pressure (mmHg)	<0.001	0.03	0.01	0.005, 0.02	1.10
Spherical Refractive Error (Diopters)	<0.001	-0.55	-0.27	-0.28, -0.26	1.62
Corneal Refractive Power (Diopters)	<0.001	-0.44	-0.28	-0.29, -0.27	1.19
Corneal Volume	<0.001	-0.04	-0.01	-0.015, -0.005	1.13
Anterior Chamber Depth (mm)	<0.001	0.20	0.48	0.42, 0.53	1.97
Anterior Chamber Angle	<0.001	0.05	0.009	0.005, 0.013	1.95
Macular Pigment Density Volume	<0.001	-0.04	-0.00002	0.000, 0.000	1.30
Peripapillary Retinal Nerve Fiber Layer Thickness	<0.001	-0.04	-0.002	-0.003, -0.001	1.09
Fundus Tessellation in the Macular Region	<0.001	0.08	0.10	0.07, 0.12	1.37
Prevalence of Myopic Maculopathy	<0.001	0.08	0.22	0.16, 0.29	1.57
Prevalence of Epiretinal Membranes	0.01	-0.02	-0.15	-0.26, -0.03	1.03
Prevalence of Pseudoexfoliation	0.007	-0.02	-0.007	-0.012, -0.002	1.09

**Fig 6:**
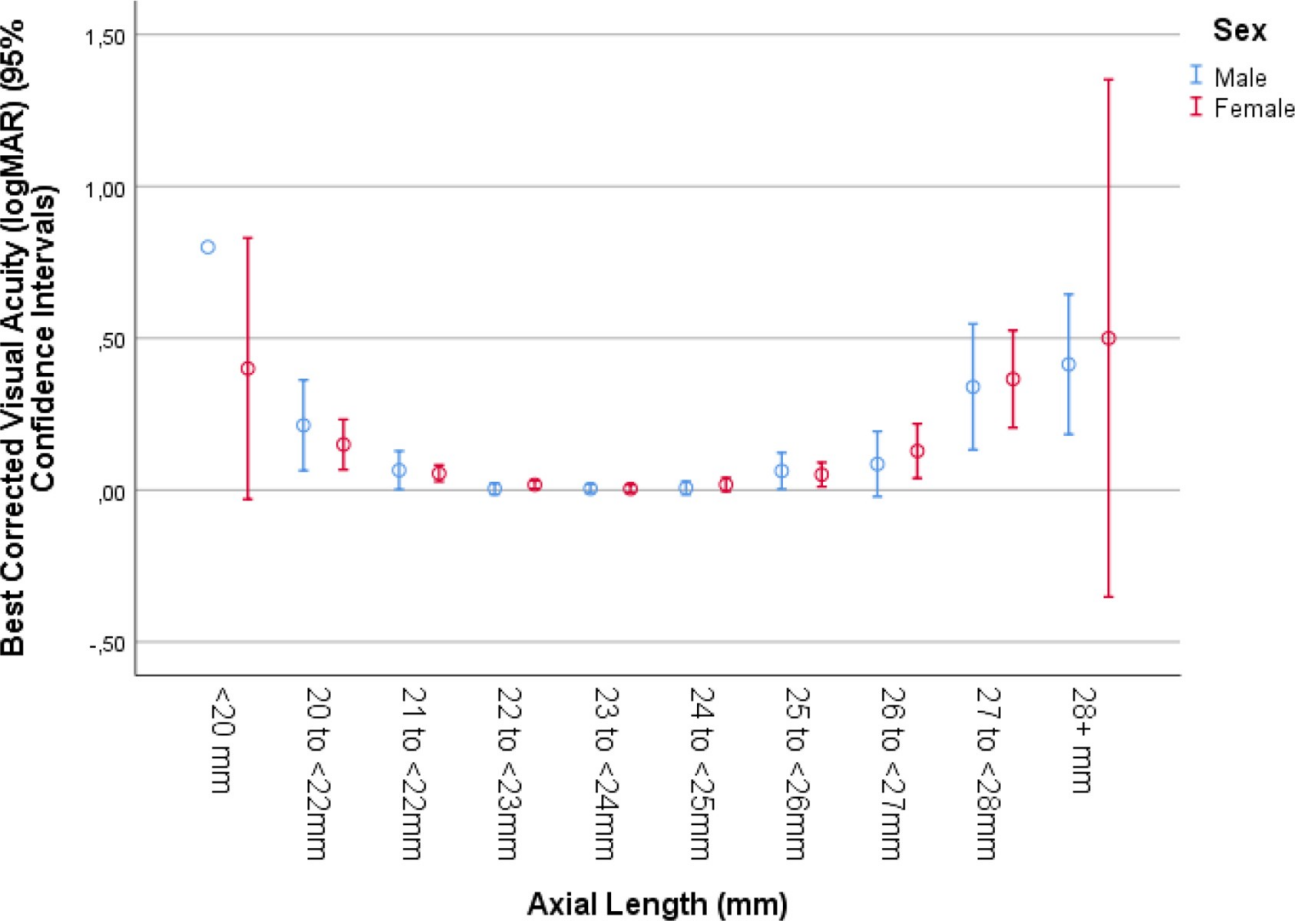
Graph showing the association between axial length and best corrected visual acuity in the Ural Eye and Medical Study, stratified by sex.

**Fig 7:**
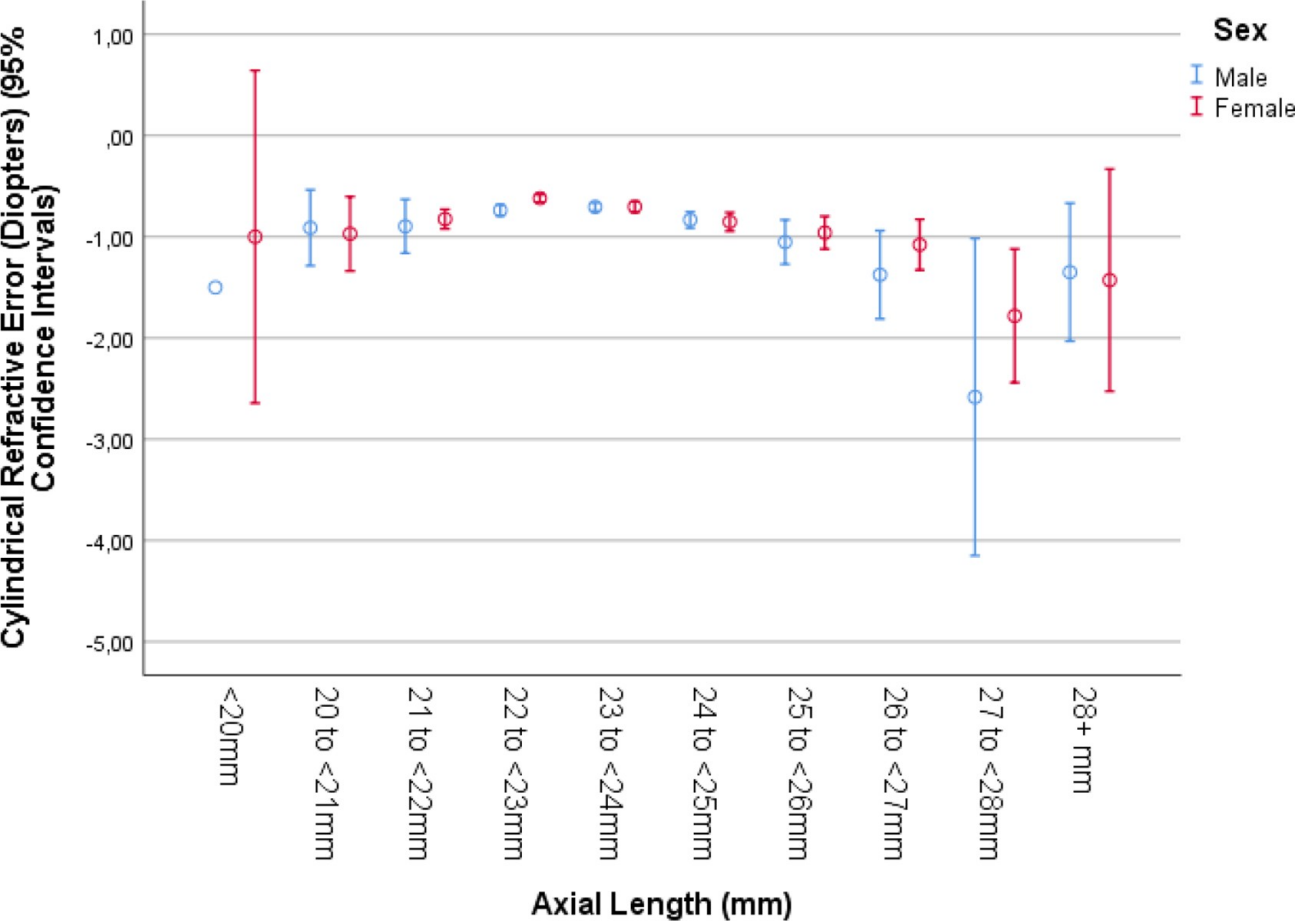
Graph showing the association between axial length and cylindrical refractive power in the Ural Eye and Medical Study, stratified by sex.

When best corrected distant visual acuity was added again to the list of independent variables, it showed a significant association with axial length (*P*<0.001; beta: -0.04; B: -0.25; 95%CI: -0.37, -0.11). In a univariate analysis, best corrected visual acuity showed a curvilinear relation with axial length, with an improvement from short axial length to medium axial length values, and again deterioration towards long axial length data (Fig 6). When cylindrical refractive error was re-added to the model, it also showed (expressed in negative diopter values) a significant association with axial length (*P*<0.001; beta: 0.05; B: 0.08; 95%CI: 0.05, 0.11). In a univariate analysis, cylindrical refractive error had a curvilinear relationship with axial length, with an increase (expressed in negative diopter values) from short axial length to medium axial length values, and again a decrease towards long axial length data (Fig 7). When body length was replaced by gender, longer axial length was associated with male sex (*P*<0.001; beta: 0.05; B: 0.10; 95%CI: 0.06, 0.14).

## Discussion

In our population-based study of an urban and rural population in Russia, the mean axial length was 23.30 ± 1.10 mm. Longer axial length was associated with older age, taller body height, higher prevalence of arterial hypertension and higher level of education, and with higher intraocular pressure, lower corneal refractive power, deeper anterior chamber depth and wider anterior chamber angle, lower macular density volume, thinner peripapillary retinal nerve fiber layer thickness, higher degree of macular fundus tessellation, higher prevalence of myopic maculopathy, lower prevalence of epiretinal membranes, and lower prevalence of pseudoexfoliation. It was not related with the prevalence of age-related macular degeneration, diabetic retinopathy, region of habitation and ethnicity. Best corrected distant visual acuity and cylindrical refractive error showed a curvilinear relation with axial length, with an improvement of best corrected visual acuity and a decrease in cylindrical refractive error from short axial length to medium axial length values, and again deterioration of best corrected visual acuity and an increase in cylindrical refractive error towards long axial length data.

These findings are in agreement with observations made in previous studies on other ethnicities, and they expand the existing knowledge of associations of axial length with ocular and systemic parameters. The mean axial length of 23.30 ± 1.10 mm in our study population was longer than the mean axial length of 22.6 ± 0.91 mm in the Central India and Medical Study, the mean axial length of 22.76 ± 0.78 mm in a rural South Indian population study, and the mean axial length of 23.1 ± 1.2 mm in a Mongolian population [27–29]. The axial length measured in our study was comparable to the data obtained in Singapore in the Tanjong Pagar Study (23.2 ± 1.2 mm), in the Los Angeles Latino Eye Study (23.4 ± 1.1 mm), and in the Beijing Eye Study (23.25±1.14 mm) [30–33]. Correspondingly, the prevalence of minor myopia (10.5%; 95%CI: 9.7, 11.3), moderate myopia (9.7%; 95%CI: 9.0, 10.5) and high myopia (1.4%; 95%CI: 1.1, 1.7) as found in our study population was comparable to the figures of the Beijing Eye Study [33].

Longer axial length was associated with correlated with taller body height in the multivariate analysis. It reflects a general tendency that taller subjects have larger eyes, as has also been shown in the Singaporean Tanjong Pagar Study, the Icelandic Reykjavik Eye Study, the Burmese Meiktila Eye Study and the Beijing Eye Study [30,33–35]. Combining the associations between longer axial length, deeper anterior chamber and taller body height, and taking a shallow anterior chamber as surrogate for an increased risk of primary angle-closure glaucoma, explains the observation made in a previous study that subjects with the prevalence of primary angle-closure glaucoma was associated with shorter body stature [36].

The correlation of longer axial length with a higher level of education as found in our study population is in agreement with all the results of previous population-based studies in which axial myopia was correlated with a higher level of education [27,31]. It is paralleled by the marked increase in the prevalence of axial myopia in the young generation in East Asia with the myopia prevalence being associated with more time spent indoors than outdoors and a higher versus lower school type [3].

Axial length increased with older age in our study population. A similar results was obtained in the Beijing Eye, the Central India and Medical Study and in a Mongolian study [19,21,25]. In several other studies, axial length decreased with older age or was independent of age [29,30,32]. Interestingly, longer axial length was associated with higher intraocular pressure in the multivariate analysis. This finding agrees with the observation made in the population-based Japanese Kumejima study in which higher intraocular pressure was significantly associated longer axial length (*P*<0.02), after adjusting for age, body mass index, systolic blood pressure, history of diabetes mellitus, thicker central corneal thickness and steeper corneal curvature [37]. In a similar manner, the Tajimi Study revealed an association between higher intraocular pressure and higher myopia after adjusting for younger age, higher body mass index, higher mean blood pressure, history of diabetes, thicker cornea and steeper corneal curvature [38]. Also in the Chinese Handan Study, higher intraocular pressure was correlated with higher myopia after adjusting for younger age, female sex, presence of diabetes mellitus, higher blood pressure, higher body mass index and thicker central cornea [39]. It was in contrast to the Los Angeles Latino Eye Study in which intraocular pressure was not related with axial length [40]. The reasons for the potential association between axial length and higher intraocular pressure have remained unclear yet. It has also remained elusive, whether higher intraocular pressure led to longer axial length or whether axial elongation-associated factors led to an increase in intraocular pressure.

The correlation of longer axial length with a deeper anterior chamber and a wider anterior chamber angle fit with the results of previous studies on that topic. It is explained by the larger inner volume of an axially elongated eye. In a similar manner, the association between longer axial length and lower corneal refractive power has also been described in previous investigations on other ethnicities [27]. The correlation between longer axial length and higher degree of macular fundus tessellation corresponds to the associations between longer axial length and thinner subfoveal choroid with a thinner subfoveal choroid being strongly associated with a higher degree of fundus tessellation [41].

Interestingly, axial length was not related with the prevalence of age-related macular degeneration. It contradicted the result of the Rotterdam Study, the French DMLA Study, the Age-Related Eye Disease Study Research, the Central India Eye and Medical Study and the Beijing Eye Study [42–46]. In the univariate analysis in our study however, longer axial length was correlated with a lower prevalence of early age-related macular degeneration (*P* = 0.001) and marginally significantly with a lower prevalence of intermediate stage of age-related macular degeneration (*P* = 0.07). A previous clinical study had suggested that the larger intraocular volume in axially elongated eyes was associated with lower intraocular concentrations of vascular endothelial growth factor and that this association might be the reason for a lower prevalence of age-related macular degeneration in myopic eyes [47].

Limitations of our study should be mentioned. First, we performed numerous statistical comparisons, first in univariate analysis and then in a multivariate analysis. Since the results of the univariate analysis only served as basis for the multivariate analysis, we did not correct for performing multiple statistical comparisons by performing Bonferroni´s correction. Although it may not be necessary to carry out a Bonferroni correction for a multivariate analysis, since the latter by itself assesses inter-dependencies between the independent parameters, most of the correlations would have remained statistically significant, if a Bonferroni correction had been applied (Table 4). Third, intraocular pressure was measured using a non-contact tonometer, what may result in different measurements as if measured by Goldmann applanation tonometry as the gold standard. Fourth, any associations between axial length and glaucoma were not assessed since that topic is analyzed in a separate investigation focused on glaucoma. Strengths of the Ural Eye and Medical Study were that the study population size was relatively large, and that the study is so far the investigation with the highest number (n = 141) of ocular parameters and diseases and of systemic parameters and diseases which were correlated with the outcome parameter of axial length. The high number of covariates reduced the risk of a confounding effect, since due to the high number of covariates, a potentially confounding factor was likely to be included in the statistical analysis.

In conclusion, in this rural an urban, multi-ethnic, typically Southern Russian study population the mean axial length was 23.30 ± 1.10 mm. This value was longer than those for a rural Central Indian population, a South Indian population and a Mongolian population and it was comparable with the data obtained in populations in Singapore, Beijing and a Latino group in Los Angeles. Longer axial length was associated with older age, taller body height, higher prevalence of arterial hypertension and higher level of education, and with higher intraocular pressure, lower corneal refractive power, deeper anterior chamber depth and wider anterior chamber angle, thinner peripapillary retinal nerve fiber layer thickness, higher degree of macular fundus tessellation, higher prevalence of myopic maculopathy, lower prevalence of epiretinal membranes, and lower prevalence of pseudoexfoliation. It was not related with the prevalence of age-related macular degeneration, diabetic retinopathy, region of habitation and ethnicity. Best corrected distant visual acuity and cylindrical refractive error showed a curvilinear relation with axial length, with an improvement of best corrected visual acuity and a decrease in cylindrical refractive error from short axial length to medium axial length values, and again deterioration of best corrected visual acuity and an increase in cylindrical refractive error towards long axial length data.

## Supporting information

S1 dataset The microdata are available in the datafile: “UEMS-2019-01-11-10-Anonymized”. (SAV)
